# Characterization of transgene expression and pDNA distribution of the suctioned kidney in mice

**DOI:** 10.1080/10717544.2017.1333171

**Published:** 2017-06-06

**Authors:** Natsuko Oyama, Yuki Fuchigami, Shintaro Fumoto, Megumu Sato, Masayori Hagimori, Kazunori Shimizu, Shigeru Kawakami

**Affiliations:** a Graduate School of Biomedical Sciences, Nagasaki University, Nagasaki, Japan;; b Graduate School of Engineering, Nagoya University, Nagoya, Japan

**Keywords:** Gene delivery, kidney, naked pDNA, spatial distribution, fibroblast

## Abstract

We have previously developed an efficient and safe transfection method for the kidney in mice: renal suction-mediated transfection. In this study, we verified the detailed characteristics of transgene expression and plasmid DNA (pDNA) in mice to develop therapeutic strategies and application to gene function analysis in the kidney. After naked pDNA was administered intravenously, the right kidney was immediately suctioned by a tissue suction device. We examined the spatial distribution of transgene expression and pDNA in the suctioned kidney using tissue clearing by CUBIC, Clear^T2^, and Sca*l*e SQ reagents. Spatial distribution analysis showed that pDNA was transfected into extravascular cells and sufficiently delivered to the deep renal cortex. In addition, we revealed that transgene expression occurred mainly in peritubular fibroblasts of the suctioned kidney by tissue clearing and immunohistochemistry. Next, we confirmed the periods of pDNA uptake and activation of transcription factors nuclear factor-κB and activator protein 1 by luciferase assays. Moreover, the use of a pCpG-free plasmid enabled sustained transgene expression in the suctioned kidney. In conclusion, analyses of the spatial distribution and immunostaining of the section suggest that pDNA and transgene expression occurs mainly in peritubular fibroblasts of the suctioned kidney. In addition, we clarified some factors for efficient and/or sustained transgene expression in the suctioned kidney.

## Introduction

The kidney is one of the most important organs to maintain life by filtrating and removing waste. Among kidney dysfunctions, accelerated progression of glomerular (Huang et al., 2014; Weins et al., 2015) and tubular injury (Fukagawa et al., 1999; Schelling, 2015) causes renal fibrosis characterized by glomerulosclerosis and tubulointerstitial fibrosis. Irreversible renal fibrosis is a common feature of end-stage renal disease (ESRD) (Duffield, 2014). Therefore, therapeutic approaches for ESRD are limited to renal replacement therapy or transplantation.

To develop a curative treatment for renal fibrosis, *in vivo* transfection methods of genes and nucleic acids into the kidney have been studied, such as viral vectors, cationic liposomes, and bubble liposomes with ultrasound as physical stimuli (Fumoto & Kawakami, 2014; Kurosaki et al., 2014). Among these transfection methods, naked plasmid DNA (pDNA) is safe and simple to use, but there are few transfection methods using pDNA because of the complexity of the renal structure and lack of specific ligands (Mukai et al., 2008; Barrera-Chimal et al., 2014).

In our previous study, we reported that direct pressure to a tissue facilitates tissue-specific and effective transfection in a tissue pressure-mediated transfection (tissue pressure method) (Mukai et al., 2008). Recently, we developed a tissue suction-mediated transfection method (renal suction method) using a tissue suction device to transfect naked pDNA into the kidney more specifically and simply (Shimizu et al., 2012, 2014; Taniguchi et al., 2016). Compared with other transfection methods in the kidney, the renal suction method may be useful in terms of safety and ease of use in mice. In addition, our suction device can be mounted to the top of an endoscope (Konishi et al., 2009; Shimizu et al., 2012). Therefore, the surgical area can be minimalized for animal scale-up to apply to clinical settings.

To develop a novel therapeutic strategy for gene therapy and application to gene functional analysis, the characteristics of transgene expression achieved by the renal suction method should be clarified in the kidney in terms of the transfected cells and underlying mechanism. Because the kidney has a complex structure consisting of abundant vasculature and multiple cell types, it is difficult to analyze the features of transfected cells. So far, transgene expression and pDNA distribution have been evaluated by the tissue section method that can offer only fragmental information and that causes the loss of spatial information. To detect pDNA and transgene expression more effectively, three-dimensional (3D) imaging is suitable (Oldham et al., 2008; Tainaka et al., 2014; Fumoto et al., 2016). Although tissue clearing methods have been used in the fields of anatomy and pathology of the kidney (Lee et al., 2014; Tainaka et al., 2014), few approaches focus on spatial evaluation of gene delivery in the kidney. Therefore, we applied 3D imaging by tissue clearing to evaluate the spatial distributions of pDNA and transgene expression in the kidney using the renal suction method. We employed three types of tissue clearing reagents for different purposes, CUBIC (Tainaka et al., 2014), Clear^T2^ (Kuwajima et al., 2013), and Sca*l*e SQ (Hama et al., 2015). CUBIC was used to clarify the dispersibility of transgene expression because it allows deep imaging compared with other tissue clearing reagents (Fumoto et al., 2016). Clear^T2^ and Sca*l*e SQ were selected to verify the distribution relationship with blood vessels because they allow staining of blood vessels by carbocyanine dye (Li et al., 2008; Kuwajima et al., 2013; Hama et al., 2015). We have previously demonstrated that pressure as a physical stimulus induces transient cellular membrane permeation and activation of nuclear factor (NF)-κB and activator protein 1 (AP-1) *in vivo* using the tissue pressure method (Mukai et al., 2010). Because temporary deformations of tissue by pressure and suction are considered to be similar (Shimizu et al., 2012; Fumoto & Kawakami, 2014), we hypothesized that the same phenomena would occur. However, there are few reports about the renal suction method. Therefore, we evaluated the periods of transient cellular uptake and activation of transcription factors NF-κB and AP-1 by the renal suction method. Moreover, we evaluated sustained transgene expression of a CpG-free plasmid transfected by the renal suction method.

## Materials and methods

### pDNA

pCMV-Luciferase (pCMV-Luc) was constructed previously (Kawakami et al., 2000). pZsGreen1-N1 and pDNAs of Mercury Pathway Profiling Luciferase system 1 (pTAL-Luc, pAP-1-Luc, and pNF-κB-Luc) were purchased from Clontech (Takara Bio Inc., Shiga, Japan). pCpG-free-LacZ and pCpG-free-Lucia were purchased from Invivogen (San Diego, CA). pCMV-Luc, pZsGreen1-N1, and pDNAs of Mercury Pathway Profiling Luciferase system 1 were amplified and purified as described previously (Kawakami et al., 2000). For amplification of the pCpG-free-plasmid (pCpG-free-LacZ and pCpG-free-Lucia), the *E. coli* competent strain GT115 (Invivogen) was used. Cy5-labeled pCMV-Luc and pZsGreen1-N1 were prepared using a Label IT Nucleic Acid Labeling Kit (Mirus Co., Madison, WI) according to the manufacturer’s protocol.

### Animals

ICR female mice (5 weeks old) were purchased from Kyudo Co., Ltd. (Kumamoto, Japan) or CLEA Japan, Inc. (Shizuoka, Japan). The mice were housed in an air-conditioned room (23 ± 3 °C) with free access to a standard laboratory diet and tap water. All animal experiments were conducted in accordance with the Guidelines for Animal Experimentation of Nagasaki University and approved by the Institutional Animal Care and Use Committee of Nagasaki University (approval number: 1308051086-6).

### Tissue suction device and modification of the suction pressure-controlled system

A suction pressure-controlled system was used with a slight modification for simple use in animal experiments. The difference from the previous system (Shimizu et al., 2012, 2014; Taniguchi et al., 2016) was the absence of a computer system that controlled the parameter of suction pressure strictly. More precisely, the electropneumatic regulator was replaced with a manual regulator (IRV20-C06G; SMC Corp., Tokyo, Japan).

### *In vivo* transfection by the renal suction method

Mice were anesthetized with 50 mg/kg sodium pentobarbital (Somnopentyl^®^; Kyoritsu Co., Tokyo, Japan), and the right kidney was exposed after laparotomy. One-hundred micrograms of pDNA in 200 μL saline was injected into the tail vein of the mice, and the right kidney was immediately suctioned at −30 kPa using the suction device. To examine the pDNA distribution, 10 μg Cy5-labeled pCMV-Luc and pZsGreen1-N1 each in 200 μL saline (diluted with unlabeled pCMV-Luc and pZsGreen1-N1 to a total of 100 μg pDNA) was injected.

### Real-time PCR analysis of *c-fos* and *c-jun*

To evaluate the activities of transcriptional factors, mRNA levels of *c-fos* and *c-jun* were measured by real-time PCR as described previously (Mukai et al., 2010) except for the extraction process and cycling conditions. Total mRNA was extracted from the right kidney using an RNeasy^®^ Mini Kit (QIAGEN, GmbH, Hilden, Germany). The cycling conditions were as follows: initial denaturation at 95 °C for 30 s, followed by 50 cycles at 95 °C for 5 s and 60 °C for 30 s. Gene-specific fluorescence was measured at 60 °C. The primers for *c-fos*, *c-jun*, and *GAPDH* were the same as those in a previous report (Mukai et al., 2010). Data are shown as relative mRNA levels to *GAPDH* mRNA levels.

### Luciferase assay

A luciferase assay of the tissue was performed as described previously (Kawakami et al., 2000). Luciferase activities were measured by a Luminometer Lumat LB 9506 (EG&G Berthold, Bad Wildbad, Germany). Each result is shown as the total amount of luminescence for 10 s (relative light unit; RLU) per gram of tissue. In the case of serum, blood samples were collected via the tail vein of mice at the indicated days. The blood samples were placed on ice for 30 min and then centrifuged at 2720 *×* *g* for 5 min at room temperature (RT). The secreted luciferase activity of 10 μL supernatant was measured using 50 μL Quanti-Luc™ (Invivogen). Obtained data are shown as RLU per mL of serum.

### X-gal staining of renal sections

Mice were sacrificed at 24 h after transfection using the renal suction method. Tissues were fixed with 0.5% glutaraldehyde in PBS containing 0.2% of 1 M MgCl_2_. Frozen sections of kidney were prepared at 24 h after renal suction transfection of pCpG-free-LacZ. The sections were washed in PBS and then stained for 4 h at 37 °C using X-gal reagent from a LacZ Tissue Staining Kit (Invivogen). The stained sample was observed under bright field using an AxioVert. A1 microscope (Carl Zeiss, Oberkochen, Germany).

### DiI staining of renal sections

At 10 min after renal suction transfection of Cy5-labeled pCMV-Luc, organs were fixed and frozen sections of the kidney were prepared. Cellular membranes were stained with 0.4 mg/mL of the lipophilic carbocyanine dye DiI (42364; Sigma Aldrich, Inc., Saint Louis, MO) for 20 min at RT, and then nuclei were counterstained with 0.5 μg/mL 4′-6-diamidino-2-phenylindole ((DAPI, D9542; Sigma Aldrich) in PBS for 15 min at RT. The stained sections were sealed with Slow Fade Gold (Thermo Fisher Scientific, San Jose, CA). Images were obtained by confocal laser scanning microscopy (CLSM, LSM710; Carl Zeiss Microimaging GmbH, Jena, Germany).

### Immunohistochemistry

Frozen sections of the kidney were prepared at 24 h after renal suction transfection of pZsGreen1-N1. After washing in PBS, the sections were blocked in 5% normal goat serum (G9023, Sigma Aldrich) for 30 min at RT. Then, the samples were incubated with primary antibodies against CD31 (1:100 dilution, #14-0311; eBioscience Inc., San Diego, CA), CD73 (1:100 dilution, 550738; BD Biosciences, San Jose, CA), or platelet-derived growth factor receptor (PDGFR) β (1:100 dilution, #14-1402; eBioscience Inc.) for 3 h at 37 °C. After washing with PBS, the samples were reacted with an Alexa Fluor 647-conjugated secondary antibody (1:200 dilution, 112-605-167; Jackson Immuno Research Laboratories, Inc., West Grove, PA) for 3 h at 37 °C. The stained sections were observed by CLSM.

### Tissue clearing method

To evaluate the transgene expression distribution, CUBIC was used. Briefly, at 24 h after transfection of pZsGreen1-N1, mice were perfused with 4% paraformaldehyde in PBS. Then, the kidney was immersed in CUBIC reagent as described previously (Tainaka et al., 2014). To visualize blood vessels with transgene expression or pDNA, Clear^T2^ (Kuwajima et al., 2013) and Sca*l*e SQ (Hama et al., 2015) reagents were used. Before immersion in Clear^T2^ and Sca*l*e SQ reagents, mice were perfused with a DiI solution according to the procedure of Li et al. (2008). To observe fluorescent-labeled pDNA, mice were perfused after 10 min or 24 h. The cleared kidney was observed by CLSM. Z-stack images were obtained as described previously (Fumoto et al., 2016).

### Statistical analysis

Statistical significance among different groups was determined by analysis of variance and the Tukey test. *p* < .05 was considered to be statistically significant.

## Results

### Distribution of pDNA using the renal suction method

#### Cellular uptake of fluorescent-labeled pDNA in tissue sections

The cellular uptake of pDNA was confirmed by the pDNA distribution in frozen sections. Ten minutes after renal suction-mediated transfection, Cy5-labeled pDNA was widely distributed in the renal cortex. In the upper part of the cortex, Cy5-labeled pDNA was mainly observed in the cytoplasm of extravascular cells and in the vascular cavity, including glomeruli, stained with DiI. In the deeper part of the cortex, most Cy5-labeled pDNA was observed in the lumen of proximal tubules and cytoplasm of interstitial cells (Figure 1(A)). Conversely, the fluorescent signals of pDNA in the renal medulla were slight. They were mainly observed around distal tubules. In both the cortex and medulla, nuclear translocation after pDNA uptake was almost absent. In the sham-operated group, Cy5-labeled pDNA was mainly distributed in blood vessels (Figure 1(A)). Some pDNA was observed in the lumen of proximal tubules and the interstitial region. In contrast, there was no detectable pDNA signal in the renal medulla.

Figure 1.Distribution of pDNA in tissue sections of the suctioned kidney determined by tissue section and tissue clearing using Sca*l*e SQ reagent. (A) Ten micrograms of Cy5-labeled pCMV-Luc was injected, and then the right kidney was suctioned at −30 kPa. Sham-operated mice were injected with the same amount of Cy5-labeled pCMV-Luc but not suctioned. After 10 min, frozen sections were prepared and stained with DiI and DAPI. The stained sections were observed by confocal laser scanning microscopy (CLSM). Magnification: ×40. Left: sham-operated group; right: renal suction group. Green: Cy5-labeled pCMV-Luc; red: DiI-stained cytoskeleton; blue: nucleus. White arrows show the uptake of Cy5-labeled pCMV-Luc to the cytoplasm of interstitial cells. All scale bars represent 50 μm. (B) Ten micrograms of Cy5-labeled pZsGreen1-N1 was injected, and then the right kidney was suctioned at −30 kPa. Sham-operated mice were injected with the same amount of Cy5-labeled pZsGreen1-N1 but without suction. After 10 min, mice were perfused with a DiI solution to stain vascular vessels. Then, the collected kidney was cleared by immersion in Sca*l*e SQ reagent. The stained kidney was observed by CLSM. Magnification: ×40. Left: Sham-operated group; right: renal suction group. Green: Cy5-labeled pZsGreen1-N1; red: vascular vessel stained by DiI; blue: nucleus. All scale bars represent 50 μm. (C) Cy5-labeled pZsGreen1-N1 was injected in the same manner as described in (B). After 24 h, mice were perfused with the DiI solution to stain vascular vessels. Then, the collected kidney was cleared by immersion in Sca*l*e SQ reagent. Magnification: ×40. Yellow: ZsGreen1; cyan: Cy5-labeled pZsGreen1-N1; magenta: vascular vessel stained by DiI; blue: nucleus. White arrows of sham-operated show Cy5-labeled pZsGreen1-N1 merged with vascular vessel. White arrows of renal suction show Cy5-labeled pZsGreen1-N1 merged and ZsGreen1 expression with nucleus. Color version of this figure is available Online.

**Figure:**
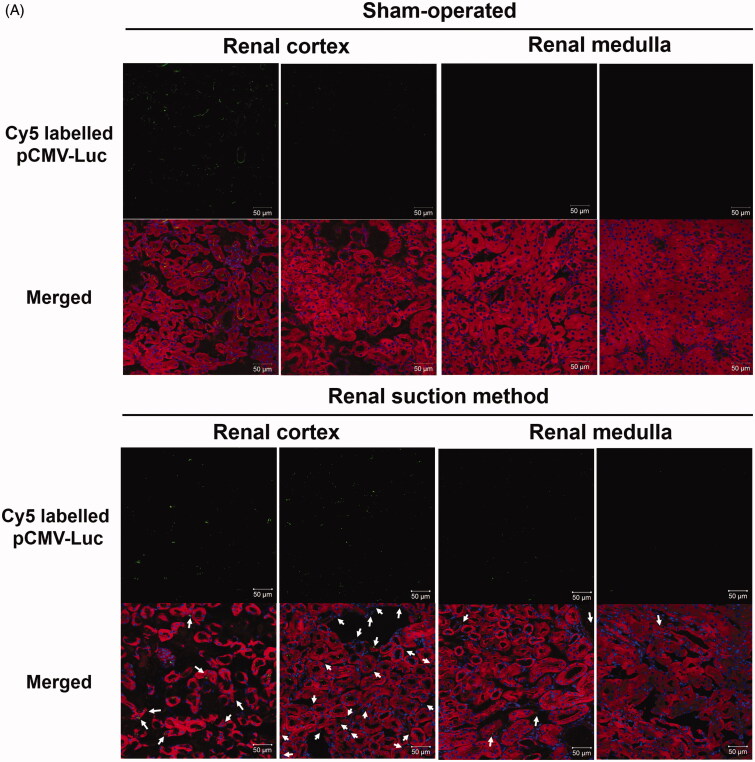

**Figure:**
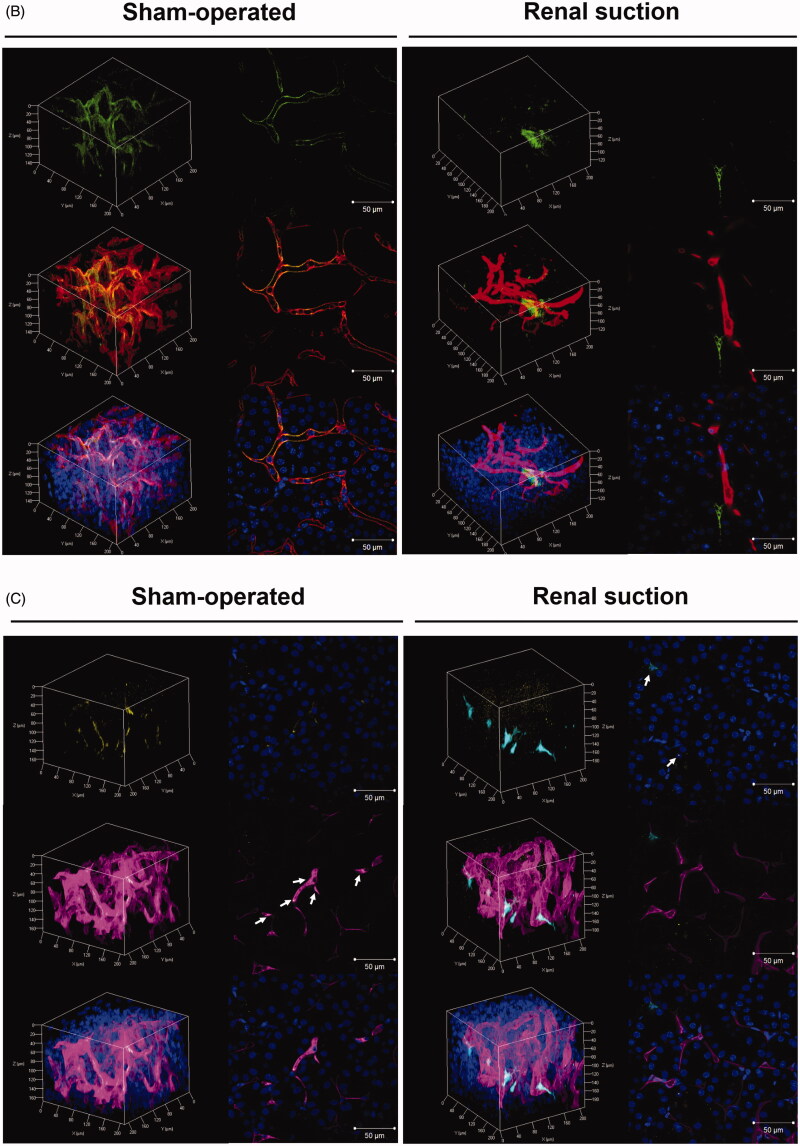

#### Spatial distribution of fluorescent-labeled pDNA determined by tissue clearin*g*

The suctioned kidney was applied to 3D vascular imaging using DiI and Sca*l*e SQ, and the distribution of pDNA was evaluated. Ten minutes after renal suction-mediated transfection, Cy5-labeled pDNA was widely distributed around renal capillaries (Figure 1(B)). Twenty-four hours after renal suction-mediated transfection, both transgene expression and nuclear translocation of pDNA were observed at the same time (Figure 1(C)). In contrast, the localization of Cy5-labeled pDNA was merged with vascular vessels at both 10 min and 24 h after transfection in the sham-operated group (Figure 1(B,C)).

### Distribution of transgene expression using the renal suction method

#### Evaluation of the transfected region and cells in tissue sections

**Figure 2.**
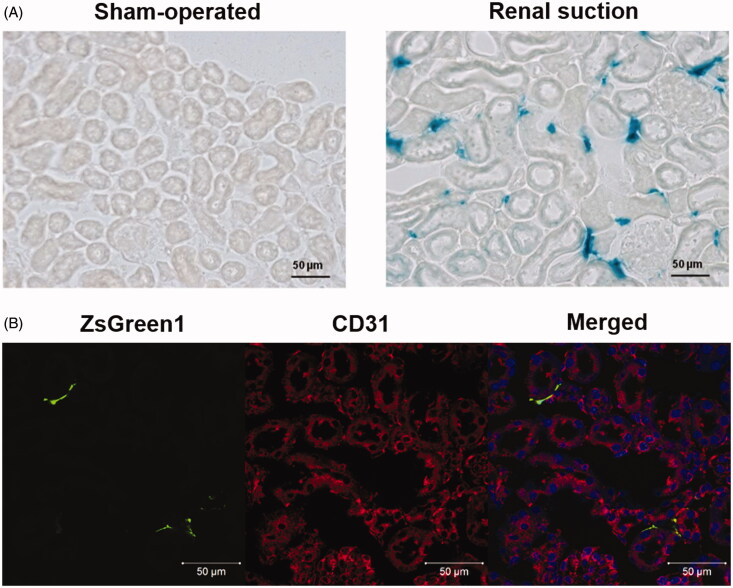
Evaluation of the transgene expression site by X-gal staining and immunostaining of frozen suctioned kidney sections. (A) The right kidney was suctioned at −30 kPa after i.v. injection of pCpG-free-LacZ. Sham-operated mice were injected with the same amount of pCpG-free-LacZ but not suctioned. The kidney was collected at 24 h after transfection, and 10 μm-thick frozen sections were stained with X-gal reagent. Left: sham-operated group; right: renal suction group. Magnification: ×20. All scale bars represent 50 μm. (B) The right kidney was suctioned at −30 kPa after i.v. injection of pZsGreen1-N1. The kidney was collected at 24 h after transfection, and 10 μm-thick frozen sections were stained with an anti-CD31 antibody and DAPI. The stained sections were observed by CLSM. Magnification: ×40. Red: CD31-positive (+) endothelial cell; green: ZsGreen1; blue: nucleus. All scale bars represent 50 μm. Color version of this figure is available Online.

The transgene expression site was evaluated by X-gal staining of frozen sections. β-Galactosidase expression was observed around glomeruli and proximal tubules in the suctioned kidney, whereas no transgene expression was observed in the sham-operated kidney (Figure 2(A)). Next, immunostaining of vascular endothelial cells was performed using an anti-CD31 antibody. Fluorescence from pZsGreen1-N1 was not merged with CD31-positive endothelial cells (Figure 2(B)).

#### Spatial distribution of transgene expression determined by the tissue clearing method

**Figure 3.**
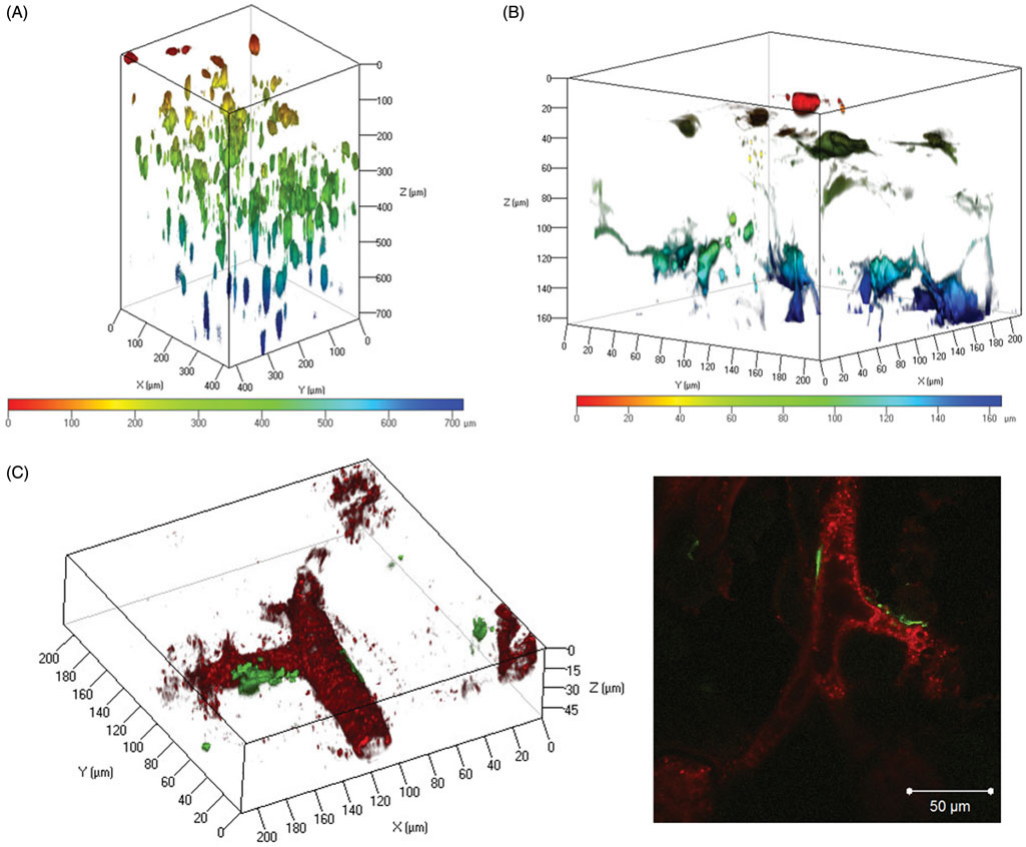
Observation of the spatial distribution of transgene expression in the suctioned kidney using tissue clearing by CUBIC and Clear^T2^. (A and B) The right kidney was suctioned at −30 kPa after i.v. injection of pZsGreen1-N1. The kidney was collected at 24 h after transfection and immersed in CUBIC reagent. ZsGreen1 expression in the cleared kidney was observed along a Z-stack by CLSM. Magnification: ×20 (A) and ×40 (B). The color chart indicates the depth on the Z-axis. (C) The right kidney was suctioned at −30 kPa after i.v. injection of pZsGreen1-N1. Twenty-four hours after transfection, the mice were perfused with a DiI solution to stain vascular vessels. Then, the collected kidney was cleared by immersion in Clear^T2^ reagent. The stained kidney was observed by CLSM. Magnification: ×40. Red: vascular vessel stained by DiI; green: ZsGreen1. Left: 3D image; right: 2D image. Scale bar in the 2D image represents 50 μm. Color version of this figure is available Online.

The spatial distribution of transgene expression in the suctioned whole kidney was examined using three tissue clearing reagents, CUBIC, Clear^T2^, and Sca*l*e SQ. Transgene expression induced by the renal suction method was observed to a depth of at least 700 μm using CUBIC reagent (Figure 3(A)). At higher magnification, the morphology of cells expressing ZsGreen1 was an elongated flat shape and consistent with that of fibroblasts (Figure 3(B)).

The suctioned kidney was applied to 3D vascular imaging using DiI and Clear^T2^ to evaluate the relationship between vascular vessels and transgene expression. ZsGreen1 was expressed on the outside of blood vessels, but not in blood vessels (Figure 3(C)).

#### Identification of transgene-expressing cells

**Figure 4.**
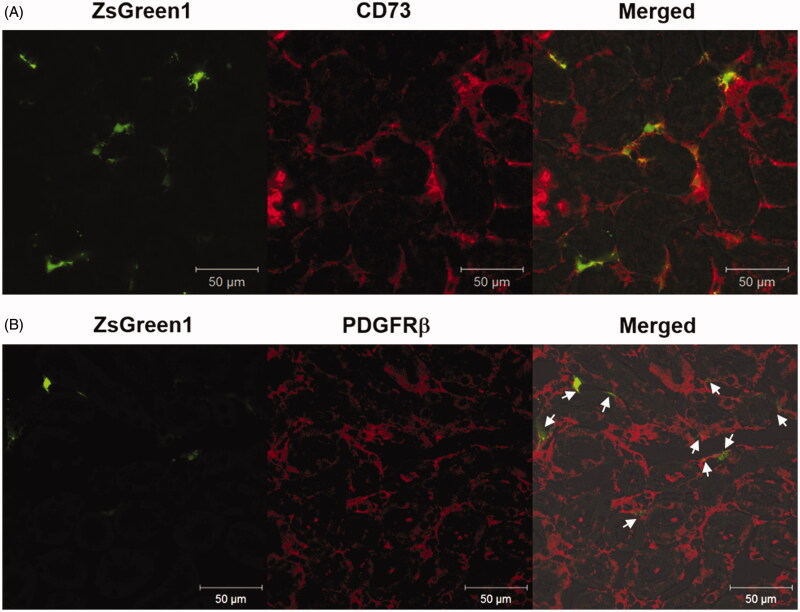
Identification of transgene-expressing cells by immunostaining of markers for peritubular fibroblasts and pericytes. (A and B) The right kidney was suctioned at −30 kPa after i.v. injection of pZsGreen1-N1. The kidney was collected at 24 h after transfection, and 10 μm-thick frozen sections were stained with anti-CD73 and -PDGFRβ antibodies. The stained sections were observed by CLSM. Magnification: ×40. Red: CD73-positive peritubular fibroblasts (A) and PDGFRβ-positive pericytes (B); green: ZsGreen1. White arrows (B) show ZsGreen1 merged with PDGFR-β-positive pericytes. All scale bars represent 50 μm. Color version of this figure is available Online.

Immunostaining of frozen sections of the suctioned kidney was performed using the perivascular fibroblast marker CD73 and pericyte marker PDGFR-β. ZsGreen1 expression was mainly co-localized with CD73-positive fibroblasts (Figure 4(A)). Conversely, some ZsGreen1 expression was co-localized with PDGFR-β-positive pericytes (Figure 4(B)).

### Periods of transient cellular uptake and activation of certain transcription factors using the renal suction method

#### Periods of cellular uptake of pDNA after renal suction stimulus

**Figure 5.**
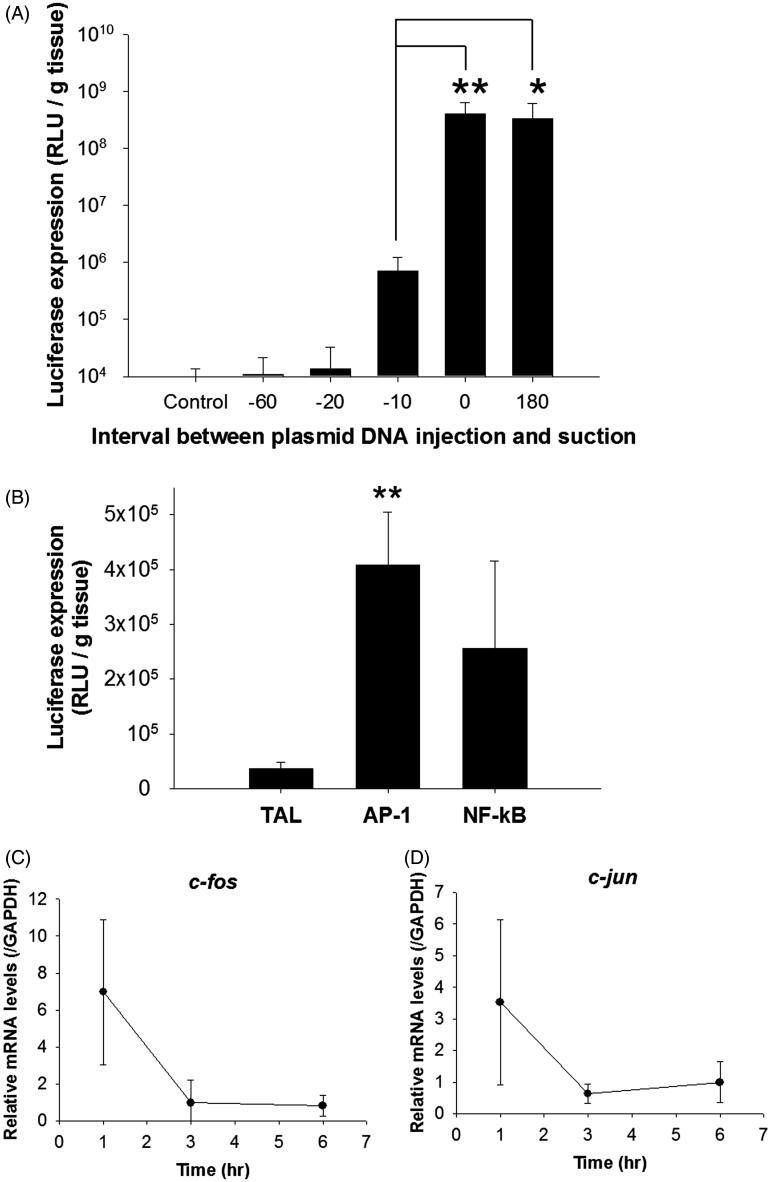
Influence of cellular uptake and transcription processes on transgene expression using the renal suction method. (A) The right kidney was suctioned at −30 kPa at various times after i.v. injection of pCMV-Luc [from before (−) 60 to after 180 s]. Transgene expression levels in the kidney were determined by a luciferase assay at 6 h after transfection. Mice in the sham-operated group (control) were injected with the same amount of pCMV-Luc as mice in the suctioned groups. Each bar represents the mean ± SD (*n* = 4–5). *p < 0.05. (B) The right kidney was suctioned at −30 kPa immediately after i.v. injection of each pDNA (pAP-1-Luc, pNF-κB-Luc, or pTAL-Luc). Transgene expression levels in the kidney were determined by a luciferase assay at 6 h after transfection. Each bar represents the mean ± SD (*n* = 6–9). Abbreviations: TAL, pTAL-Luc; AP-1, pAP-1-Luc; NF-κB, pNF-κB-Luc administration groups. **p < .01 vs. pTAL-Luc administration group. (C and D) The right kidney was suctioned at −30 kPa immediately after i.v. injection of pCMV-Luc. The mRNA levels of (C) *c-fos* and (D) *c-jun* in the kidney were determined by real-time PCR at 1, 3, and 6 h after transfection. mRNA levels were normalized to the mRNA level of *GAPDH* in each sample (*n* = 3–5).

Transgene expression levels were evaluated at various timings of suction from 60 s before to 180 s after intravenous (i.v.) injection of pDNA. As a result, the luciferase levels immediately after (0 s) and at 180 s after pDNA injection were higher than those at any other time (Figure 5(A)). In contrast, the luciferase levels at 10, 20, and 60 s before injection were less than those in the abovementioned condition, and those at 20–60 s before were the same as the control level (Figure 5(A)).

#### Influence of the activities of transcription factors on transgene expression after renal suction stimulus

To clarify the contribution of pDNA binding to transcription factors for transgene expression in the suctioned kidney, the activities of transcription factors were evaluated. Compared with pTAL-Luc (negative control) that has no binding site for transcription factors, higher luciferase expression in the right kidney was obtained after administration of pAP-1-Luc or pNF-κB-Luc (Figure 5(B)).

In addition, at 1 h after transfection, the mRNA levels of immediate early genes of AP-1 transcriptional factor, *c-fos* and *c-jun*, were increased by about 7- and 3.5-fold, respectively (Figure 5(C,D)). At 3 h after transfection, the levels of both mRNAs had decreased to normal levels.

### Duration of transgene expression using the renal suction method

**Figure 6.**
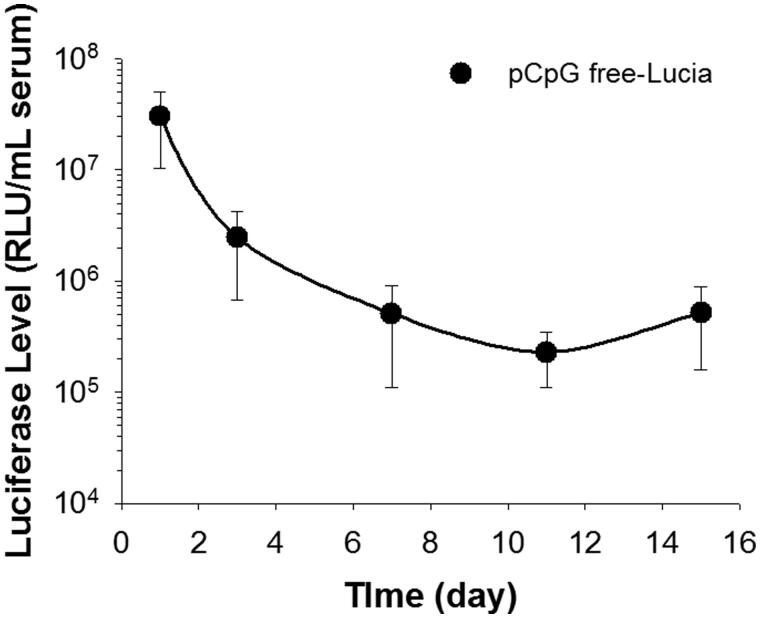
Duration of transgene expression after pCpG-free plasmid transfection by the renal suction method. The right kidney was suctioned at −30 kPa. Transgene expression levels in serum were determined by luciferase assays at 1, 3, 7, 11, and 15 days after transfection. Data points represent means ± SD (*n* = 5).

The duration of transgene expression from each plasmid vector was determined at the serum level. The transgene expression of pCMV-Luc was reduced to an undetectable level at 1 week (data not shown). However, the transgene expression of pCpG-free-Lucia continued for at least 2 weeks in serum (Figure 6).

## Discussion

In present study, characterization of the renal suction method was performed in mice. Using three tissue clearing reagents, CUBIC, Clear^T2^, and Sca*l*e SQ, we clarified the spatial distribution of pDNA and transgene expression in the kidney using the renal suction method (Figures 1(B,C) and 3). Combination with vascular staining can offer supplemental information about transfected cells and cellular uptake or nuclear translocation of pDNA (Figures 1(B,C) and 3(C)). Therefore, our results indicate that evaluation of the spatial distribution is a promising approach to verify the characterization of gene delivery systems more effectively in the kidney.

In terms of the diversity of therapeutic cells in the kidney, the transfected region and cells need to be determined for development of a novel therapeutic strategy. The results of X-gal staining suggested that pDNA was transfected into renal capillaries and the interstitial region (Figure 2(A)). In general, macromolecular extravasation from blood vessels to tissues can be restricted by vascular barriers such as tight junctions, adhesion junctions, and basement membranes (Lampugnani et al., 1993; Hu et al., 2008). In view of the fact that pDNA was injected via the i.v. route, we expected that the transfected cells should be vascular endothelial cells. Surprisingly, the results of immunostaining for CD31 revealed that the transfected cells were not endothelial cells but extravascular cells (Figure 2(B)). Evaluation using only tissue sections fails to reveal serial transgene expression in the whole kidney. Therefore, we evaluated the relationship between transgene expression and vascular vessels by the spatial distribution using the tissue clearing method. Unexpectedly, transgene expression was observed outside of blood vessels even in 3D imaging (Figures 1(C) and 3(C)). Next, we confirmed whether pDNA transfer was localized in extravascular cells. The distribution was consistent with that of transgene expression (Figure 1(B,C)). Tissue deformation induced by suction may alter endothelial cell gap formation to enable pDNA entry. Considering the fact that pericytes and fibroblasts are in close proximity to endothelial cells among extravascular cells (Smith et al., 2012; Geevarghese & Herman, 2014), the transfected cells were expected to be these cell types. We confirmed the transfected cells by immunostaining of tissue sections. Immunostaining for CD73 and PDGFR-β in suctioned kidney sections revealed that the majority of transgene expression was co-localized with CD73-positive peritubular fibroblasts (Figure 4(A)), and some transgene expression was merged with PDGFRβ-positive pericytes (Figure 4(B)). The difference between these cell types in terms of co-localization is probably attributable to the relative ratio of pericytes to endothelial cells, which is reported to be 1:2.5 in the kidney (Smith et al., 2012). Tsujie et al. have reported that AVE-type HVJ liposomes delivered via intra-ureter administration retrogradely can transfect into peritubular fibroblasts (Tsujie et al., 2000). To the best of our knowledge, this is the first report indicating that the tissue suction method via i.v. administration mainly transfers pDNA to peritubular fibroblasts. Taken together, we provide evidence that pDNA can be transfected into peritubular fibroblasts by penetrating blood vessels using the renal suction method.

We evaluated the underlying mechanism of efficient transgene expression using the renal suction method. In terms of the cellular uptake process, we have previously shown the possibility that the physical stimulus of pressure increases cell membrane permeation for about 10 s using the tissue pressure method *in vivo* (Mukai et al., 2010). In the present study, we confirmed the duration of transient permeation of cell membranes caused by the renal suction method to clarify that the stimulus of renal suction induces transient cellular uptake in the same manner as renal pressure. Cellular uptake of pDNA by the renal suction method occurred within 10 s and was almost undetectable over 20 s (Figure 5(A)). This result agrees with that of the tissue pressure method (Mukai et al., 2010). In addition, we imaged fluorescent-labeled pDNA to obtain more information about the cellular uptake of pDNA. The results obtained from tissue sections revealed that Cy5-labeled pDNA was widely distributed in the renal cortex and slightly detected in the medulla at 10 min after renal suction-mediated transfection (Figure 1(A)). This result is also congruent with our previous report of the renal pressure method (Mukai et al., 2010), and it would be caused by the abundance of capillaries in the renal cortex compared with the renal medulla. More precisely, the uptake of Cy5-labeled pDNA had mainly occurred in extravascular cells at 10 min after renal suction-mediated transfection (Figure 1(A,B)). The deformation of tissue induced by the suction device can also cause a transient increase of cell membrane permeation in extravascular cells within 10 s in mice.

Some transcription factors are reported to enhance intracellular trafficking and nucleus entry by binding to pDNA (van Gaal et al., 2011; Badding et al., 2012). Among them, rapid and transient activation of AP-1 and NF-κB have reported in response to a wide range of stimuli (Fujioka et al., 2004; Sen & Smale, 2010). In a previous study, we demonstrated the involvement of NF-κB and AP-1 in efficient transgene expression *in vivo* using the tissue pressure method (Mukai et al., 2010). Furthermore, Lam et al. have reported that mechanical stress, including cyclic stretching, alters the activation and/or nuclear localization of key transcription factors including NF-κB and the *c-fos* and *c-jun* subunits of AP-1 *in vitro* (Lam et al., 2008). In this study, we confirmed that the stimulus of renal suction promotes the activation of transcription factors NF-κB and AP-1. AP-1 and NF-κB were significantly activated by renal suction stimulus (Figure 5(B)). This result is supported by the results indicating that the mRNA levels of *c-fos* and *c-jun*, which comprise AP-1, were increased transiently prior to the increase of AP-1 (Figure 5(C,D)). These results are also in accordance with those of the tissue pressure method (Mukai et al., 2010). Moreover, we visualized nuclear transportation of fluorescent-labeled pDNA by tissue clearing. Transgene expression and nuclear translocation of Cy5-labeled pDNA was observed at the same time of 24 h after renal suction-mediated transfection (Figure 1(C)). Therefore, these results support our hypothesis that the deformation of tissue by pressure and suction causes nuclear localization and activation of NF-κB and AP-1 for efficient transgene expression *in vivo*.

In addition to the cytoplasm of extravascular cells, Cy5-labeled pDNA was observed in the vascular cavity and the luminal surface of proximal tubules (Figure 1(A)). Considering that most Cy5-labeled pDNAs remained in blood vessels in the sham-operated group at each time point (Figure 1), our results suggest that pDNA passes through blood and glomerular barriers by the deformation from suction. Unexpectedly, fluorescent-labeled pDNA was attached to the vascular cavity and the lumen of renal tubules without being washed out by perfusion (Figure 1). Rombouts et al. proposed that fluorescent-labeled pDNA has a higher affinity for lipid structures, including liposomes and the endosomal membrane, by increasing the level of hydrophobicity, but the cellular uptake remains unaffected (Rombouts et al., 2014). Based on this report, the higher affinity for lipid membranes may account for the attachment of Cy5-labeled pDNA to the vascular vessels and the luminal surface of proximal tubules. Although fluorescent-labeled pDNA was distributed in these structures (Figure 1(A)), there was no transgene expression in endothelial cells or tubular epithelial cells (Figures 2 and 3). In common, these cells are continuously exposed to fluid shear stress and the apical surfaces of them are covered by dense porous surface layers: microvilli of proximal tubules and glycocalyx of endothelial cells (Weinbaum et al., 2010; Curry & Adamson, 2012). These structures can regulate its membrane transport proteins and maintain homeostasis as mechanosensors (Weinbaum et al., 2010; Curry & Adamson, 2012). Therefore, the reason why pDNA is not transfected into these cell types might be accounted for by the barrier roles of each surface layer, but further study is necessary.

Progression of tubulointerstitial fibrosis is a final common pathway to ESRD (Duffield, 2014). Myofibroblasts are well known as the main cause of progressing tubulointerstitial fibrosis, and several reports have revealed that local fibroblast expansion accounts for about 50% of the myofibroblast pool (LeBleu et al., 2013; Souma et al., 2013). Therefore, the renal suction method may be one of the best applications for treatment of tubulointerstitial fibrosis from the viewpoint of transfected cells. However, it was unclear whether this method can deliver pDNA to the deep cortex. Therefore, we evaluated the spatial distribution to clarify the depth of transgene expression. Using the renal suction method, transgene expression was observed to a depth of at least 700 μm (Figure 3(A)). The total thickness of the renal cortex in mice is reported to be about 1 mm from the surface (Zhai et al., 2003; Kobayashi et al., 2004). The depth of transgene expression using the renal suction method could be sufficient to deliver pDNA to the deep cortex of the kidney. Thus far, the renal suction method may be one of the best applications for the treatment of tubulointerstitial fibrosis.

In addition to the depth of transgene expression, the duration of transgene expression is important for efficient gene therapy because there is a limitation to the number of transfections. Several studies have reported that removal of CG dinucleotides from pDNA can achieve long-term transgene expression by avoiding the inflammatory response (Kosovac et al., 2011; Pringle et al., 2012). Therefore, we confirmed the duration of transgene expression of a CpG free plasmid encoding a secreted luciferase protein, Lucia, at the serum level. Compared with expression of pCMV-Luc at 1 week (data not shown), pCpG-free-Lucia was expressed for up to 2 weeks at the serum level (Figure 6). For clinical use, mounting the tissue suction device to an endoscope can solve the invasiveness of surgery. To date, several factors, such as hepatocyte growth factor and Klotho, have been reported to have a therapeutic potential for renal fibrosis by reducing fibrogenic cytokine production as well as fibronectin and type I collagen deposition (Dai et al., 2004; Satoh et al., 2012). Therefore, using CpG-free plasmids encoding these therapeutic genes could be effective for the treatment of renal fibrosis.

## Conclusions

We characterized transgene expression in the kidney using the renal suction method in mice. We evaluated the spatial distribution of pDNA and transgene expression of the suctioned kidney using tissue clearing reagents CUBIC, Clear^T2^, and Sca*l*e SQ. Our results using tissue clearing provide evidence that pDNA penetrates blood vessels and transfects into extravascular cells of the suctioned kidney. Based on the spatial distribution of the suctioned kidney, we succeeded in clarifying that the renal suction method mainly transfects pDNA into peritubular fibroblasts of the deep cortex by immunostaining of tissue sections. In addition, efficient transgene expression induced by the renal suction method can involve transient cellular uptake and spontaneous activation of some transcription factors in the suctioned kidney. Moreover, the use of a pCpG-free plasmid showed sustained transgene expression in the kidney using the renal suction method. These findings may facilitate development of gene therapy and gene function analysis using the renal suction method.
